# Philadelphia College of Pharmacy

**Published:** 1859-05

**Authors:** 


PHILADELPHIA COLLEGE OF PHARMACY.—The number of students in attendance at this institution during the last session was 88. The degree of Graduate in Pharmacy was conferred on 21 gentlemen. The valedictory address was delivered by Professor Bridges, well known as an able teacher and accomplished chemist.

